# Quantitative Analyses of the Tumor Microenvironment Composition and Orientation in the Era of Precision Medicine

**DOI:** 10.3389/fonc.2018.00390

**Published:** 2018-09-25

**Authors:** Florent Petitprez, Cheng-Ming Sun, Laetitia Lacroix, Catherine Sautès-Fridman, Aurélien de Reyniès, Wolf H. Fridman

**Affiliations:** ^1^INSERM, UMR_S 1138, Cordeliers Research Center, Team Cancer, Immune Control and Escape, Paris, France; ^2^University Paris Descartes Paris 5, Sorbonne Paris Cite, UMR_S 1138, Centre de Recherche des Cordeliers, Paris, France; ^3^Sorbonne University, UMR_S 1138, Centre de Recherche des Cordeliers, Paris, France; ^4^Programme Cartes d'Identité des Tumeurs, Ligue Nationale Contre le Cancer, Paris, France

**Keywords:** tumor microenvironment, prognostic markers, quantitative analysis, human cancer, immunotherapy

## Abstract

Tumors are formed by aggregates of cells of various origins including malignant, stromal and immune cells. The number of therapies targeting the microenvironment is increasing as the tumor microenvironment is more and more recognized as playing an essential role in tumor control. In the era of precision medicine, it is essential to precisely estimate the composition, organization and functionality of the individual patient tumor microenvironment and to find ways to therapeutically modulate it. To quantify the cell populations present in the tumor microenvironment, many tools are now available and the most recent approaches will be reviewed herein. We provide an overview of experimental and computational methodologies used to quantify tumor-associated cellular populations, including immunohistochemistry, flow and mass cytometry, bulk and single-cell transcriptomic approaches. We illustrate their respective contribution to characterize the microenvironment. We also discuss how these methods allow to guide therapeutic choices, in relation to the predictive value of some characteristics of the microenvironment.

## Introduction

Tumors are aggregates of cells, among which are not solely found malignant cells, but also a vast variety of other cell populations, notably immune cells of all types, blood and lymphatics vessels and fibroblasts (1). The constant dialogue between the cancer cells and the host cells composing the tumor microenvironment (TME) is the essence of several hallmarks of cancer (2). In particular, angiogenesis, tumor-promoting inflammation, and immune escape govern the composition and functional orientation of the TME. Moreover, a growing body of evidence links the infiltration of tumors by immune cells to the clinical outcome of the disease (3). With the growing importance of immunotherapies to treat cancer patients, it has become crucial to be able to decipher the composition and the functional orientation of the microenvironment. Many teams have invested efforts on developing tools to quantify the TME populations with a tremendous variety of technological approaches. Here, we review some of them, both experimental and computational, and provide insights as to how they can help achieve proper personalized medicine. Each type of experimental design presents advantages and drawbacks as compared to other settings. These are summarized in Table 1.

**Table 1:** Comparison of the main experimental methodologies that can be used to analyze the TME.

		**Number of markers**	**Throughput**	**Spatial organization**	**Precise quantification**	**Many public datasets available**
IHC	Brightfield	Low	Low	Yes	Yes	No
	Immunofluorescence	Low to medium	Low	Yes	In some settings	No
Cytometry	Flow Cytometry	Low to medium	Medium	No	Yes	No
	Mass Cytometry	Medium	Medium	No	Yes	No
Transcriptomics	RNA-Seq and micro-arrays	High	High	No	Yes	Yes
	Single-cell transcriptomics	High	High	In some settings	No	Yes

*Green, well fitted; yellow, medium; red, poorly fitted*.

## TME description by *in situ* immunohistochemical imaging

One of the most straightforward ways to analyze the TME is to use immunohistochemistry (IHC) or immunofluorescence (IF) to directly quantify various populations. Compared to other methods, IHC retains the tissue structure and therefore allows to analyze the anatomical location of cells within the tumor, as well as the detection of lymphoid-like structures or intratumoral blood vessels.

Both IHC and IF use a primary antibody to target the molecule of interest. A secondary antibody conjugated to either a catalytic agent (IHC) or a fluorophore (IF) is then used to amplify the signal and to reveal the distribution of the target molecule. These steps can be repeated to analyze different markers. The staining can be observed through a microscope or scanned images can be analyzed by histopathology software to accurately quantify each marker. Through the combination of different markers and the shape and size of cells, different cells can be quantified. A nuclear counterstain can further increase the accuracy of image analysis.

Until recently, this methodology was limited to only a small number of markers that could be assessed simultaneously, due to the cross-reactivity between primary and secondary antibodies. Therefore, the description of the TME of large series of patients was a long and complex procedure. However, recent efforts have allowed a larger number of markers that can be stained on the same slide, notably by using IF and automation, allowing up to seven colors for the same slide (4, 5). One multiplexing method is the tyramide signal amplification (TSA) system. In this system, a fluorophore-conjugated tyramide is catalyzed by horseradish peroxidase conjugated to the secondary antibody, and binds covalently around the epitope of interest. This allows both the primary and secondary antibodies to be stripped from the tissue, avoiding the risk of antibody cross-reactivity in the next staining round (6). Overall, the multiplexed analysis of several markers on the same tissue section allows for a precise estimation of co-expression of markers by the same cells, or the spatial distribution of related markers (7).

Besides traditional IHC/IF methods, other studies use metal-tagged antibodies and mass cytometry to reveal the tissue staining. These methods allow to read up to 32 markers on formalin-fixed paraffin-embedded (FFPE) tumor sections (8, 9). Such emerging systems could dramatically expand the number of markers that can be assessed simultaneously and have a huge potential for the future of TME analysis. To also detect cytokines, which are difficult to measure through traditional IHC/IF, methods have been developed to detect mRNA on FFPE slides and couple this with IHC (10–12). Such methods allowed to show that, in breast cancer, the density of CXCL10 expressing cells correlated with T cells density (13).

Several IHC-based studies reported characterizations of the TME with prognostic impact. In particular, in colorectal cancer (CRC), the Immunoscore, an aggregate measure of CD3^+^ and CD8^+^ T cells in the tumor core and the invasive margin, that yields higher significance than each region separately (14), was shown to be a stronger prognostic factor than microsatellite instability (15) and TNM staging system (14). An international consortium has recently validated this approach on a very large series of tumors (16). IHC has also been used to assess the prognostic impact of various immune cell types in virtually all non-hematologic malignancies reporting a widespread positive impact of CD8^+^ T cells on clinical outcome (3). However, there are some exceptions to this rule. For instance, the poor prognostic impact of CD8^+^ T cells in clear cell renal cell carcinoma (ccRCC) or prostate cancer was shown using IHC (17, 18). IHC is currently one of the main methods used to study and characterize tertiary lymphoid structures, local lymph node-like immune cell aggregates composed of a T cell zone with mature dendritic cells and a B cell zone (19). They can be identified by the presence of a CD20^+^ B cell aggregate surrounded by a CD3^+^ T cells aggregate containing DC-Lamp^+^ mature dendritic cells (19). They were shown to be associated to a better prognosis in a large array of cancers (19) including lung squamous cell carcinoma (20). In colorectal cancer, their prognostic impact was found to be dependent on their maturation level (21).

IHC can also help to guide therapies by identifying patients which are more likely to respond to specific treatments. In metastatic renal cell cancer, IHC-based measures of several biomarkers can help clinicians select between sunitinib or sorafenib (22). They showed that patients with high expression of CAIX, HIF-2α, and CD31 responded better to sunitinib than sorafenib, while patients with higher VEGFR1 or PDGFRB expression benefited more from sorafenib than sunitinib. The expression of PD-L1 in tumors has also been highly scrutinized to identify patients likely to respond to blockade of the PD-1/PD-L1 axis (23). In some cases, detection of PD-L1 by IHC is even a companion diagnostic assay with strong discrepancies in the methodology used (24). The use of PD-L1 as a theranostic biomarker is not as clear as expected: around 15% of patients with PD-L1-negative tumors respond to PD-1 blockade (25), and some patients with tumors with a high expression of PD-L1 fail to respond to similar treatments (23). Therefore, more complex approaches are being tested to refine the specificity and sensitivity of these tests, notably thanks to multiplexing to include T cell markers (24).

The progress of machine learning has allowed large progress to be made on automated analysis of histopathological images (26). By training complex mathematical models, often neural network on large sets of tumor slides, such approaches can identify tumor cells or asses pathological stage (27, 28), and a method directly predict patients' survival probability (29). Several such machine learning methods aim at quantifying the TME, especially lymphocytes, with performance comparable to pathologists (30). This could allow fast and reproducible analysis of TME composition on large series as well as routine quantification of tumor-infiltrating lymphocytes for many patients.

## Analysis of the TME composition and functional orientation using cytometry

To have a more precise view of the functionality of cells composing the TME, other methodologies can be used. Cytometry is one of these methods, which enables a single-cell description on several markers. To be analyzed by flow cytometry, the tissue is first dissociated to a single-cell suspension. Cells are then marked with fluorophore-conjugated antibodies for target molecules. Each cell's markers will be read with a laser, a method that can analyze thousands of events per second. Each population of interest can subsequently be analyzed by the multiparametric expression of various markers (“gating”). Cytometry presents several advantages compared to other methods: it can quantify single-cell data for large numbers of cells (usually millions) in a relatively short time frame and can identify various populations simultaneously, including rare populations. This allows to access not only the proportions of populations but also their functional orientation.

Flow cytometry has been extensively used to study tumor-infiltrating immune cells, with early description of the presence of dendritic cells for instance (31). It has notably been used to describe and characterize myeloid-derived suppressor cells (MDSC) in mouse models (32) and in human tumors (33). MDSC form a heterogeneous population of myeloid-originated cells with immuno-suppressive properties whose role in cancer raises a growing interest (34).

The relatively recent development of mass cytometry has allowed several dozens of markers to be assessed simultaneously, with cells marked by metal-tagged antibodies subsequently quantified by time-of-flight mass spectrometry (35). It has allowed detailed description of tumor-infiltrating immune cells (36). In particular, in ccRCC, mass cytometry has unveiled a wide diversity of tumor-associated macrophages and T cells (37), including 16 subsets of macrophages and 21 subsets of T cells, which allows a much more precise description of the functionality of these cells and their association with prognosis. Another study (38) focused on early lung adenocarcinoma and used mass cytometry coupled with single-cell RNA-Seq to extensively describe T cells, NK cells and myeloid cells compartments.

Flow cytometry has also been used to assess the profiles of tumor-infiltrating T cells and their correlation with prognosis or patients' response to therapy, in the primary tumor or in metastatic disease. In primary ccRCC, it was shown that tumors could be classified into three groups based on the profile of their tumor-infiltrating lymphocytes (TILs) (39). Notably, patients bearing tumors with exhausted TILs phenotypes and regulatory T cells were reported to experience early relapse. Another study showed that metastatic melanoma patients with increased PD-1 and CTLA-4 expression on tumor-infiltrating CD8^+^ T cells were more likely to respond to anti-PD-1 therapies (40). Finally, another team showed using flow cytometry that in a mouse model of lung adenocarcinoma, acquired resistance to PD-1 blockade was associated to an increased expression of other inhibitory molecules, especially TIM-3 (41).

## Estimation of the TME composition using gene expression data

The methods presented above allow for a precise identification of the immune contents of the TME, but are cumbersome to apply to large series of tumors. Several methods have been developed to mine transcriptomic data of tumors in order to decipher the TME composition (42). These methods use different approaches. Some of them are based on Gene Set Enrichment Analysis [GSEA (43)] to provide signatures of immune cells (44) or quantify TME populations, such as xCell (45) or TIminer (46), a computational framework that performs several immunogenomics analyses, including a GSEA-based quantification of the immune infiltrate.

Becht et al. reported in 2016 a novel approach using gene signatures, with a tool called MCP-counter (47). MCP-counter relies on the analysis of transcriptomic markers that are only expressed in one cell population and provides scores that are proportional to the cell proportion in the analyzed sample. Signatures are available for eight immune populations, as well as endothelial cells and fibroblasts. This approach is robust and allows to compare different samples (48).

Other methods use the deconvolution framework, that is the estimation of cell population contribution to the overall signal by solving a set of linear equations (49). Several methods are based on this approach, with various solving algorithms, such as least square regression (50), constrained least square regression (with non-negativity of cell fractions) (51), or nu-support vector regression. The latter is used by CIBERSORT (52), which proved efficient in comparing one population with another and accurately estimates 22 immune populations. Other notable deconvolution methods include TIMER (53) which performs sequential estimations to refine the estimation of six immune populations, EPIC (54), which estimates the proportion of cells that are not accounted for (among which are tumor cells) as well as the relative abundances of five tumor-infiltrating immune cell types, endothelial cells and fibroblasts, and quanTIseq (55), a deconvolution pipeline designed for raw RNA-seq data.

Finally, other methods perform complete deconvolution, that is simultaneous estimation of the cell types proportions and of their transcriptomic profiles, although generally for a limited number of populations. This is usually done using non-negative matrix factorization (56, 57). In a pioneering work, Venet et al. performed complete deconvolution on colorectal cancer transcriptomes (56). Although their method cannot be identified to quantify specific or precise subpopulations, they identified and quantified populations expressing signatures related to immune cells. Some methods, such as ISOpure (58) can, if provided with reference profiles, separate the part of the transcriptome that is related to tumor or healthy cells.

One of the main advantages of the aforementioned methods is that they can be applied to very large series of transcriptomic data, of which many are publicly available on repositories such as Genomics Data Commons for the Cancer Genome Atlas data, or Gene Expression Omnibus. This allows to draw conclusions on extensive cohorts, with more statistical power. Notably, MCP-counter was used to assess the prognostic impact of various cell types in different malignancies (47).

As another output of TIminer, Charoentong et al. designed an immunophenoscore, based on the expression of immune checkpoint genes, immune effector cells, immune suppressor cells and antigen processing and presenting machinery genes (59). They showed that the immunophenoscore was associated to survival in ccRCC, melanoma, breast and bladder cancers. It is also associated with response to CTLA-4 and PD-1 blockade in metastatic melanoma cohorts.

One of the main issues with transcriptomics methods is the loss of spatial information to localize identified cell types in the tumor. Using barcodes associated to different regions of the analyzed tissue prior to RNA-Seq, Ståhl et al. have designed a spatial transcriptomics method (60). This method can be used to infer immune cells presence and localization in tumor samples (61).

Single-cell RNA sequencing (scRNA-Seq) technologies raise a growing interest and can be a particularly valuable tool in the context of immunity (62). Indeed, analyzing immune cells at the single-cell scale allows a complete determination of the cell's phenotype and functional orientation. In tumor immunology, scRNA-Seq has been used to comprehensively characterize the TME of metastatic melanoma (63) and liver cancer (64), for instance to deeply characterize well-defined populations, such as tumor-infiltrating T cells or identify the T cell receptor repertoire (64). Interestingly, scRNA-Seq allows to study rare populations, whose expression is hard to segregate from noise in bulk transcriptomics. scRNA-Seq can also guide deconvolution by informing on the expression profiles of tumor-infiltrating immune cells (54, 65). This can alleviate a limitation of several deconvolution methods, which use cells purified from blood of healthy donors to derive transcriptomic signatures.

## Conclusion

Tumors are engaged in intricated relationships with their microenvironment. The TME is strongly variable between cancers and patients, in terms of composition and functional orientation. The interplay between a tumor and its TME has strong clinical implications, both for prognosis and treatment options, especially in the era of checkpoint blockade therapies. Therefore, studying what cell types are present in the TME and in which numbers is of tremendous importance. To do so, a large variety of methods have been developed and continuously refined. This covers immunohistochemistry, flow and mass cytometry, and the extensive use of transcriptomics, both bulk and single-cell.

These various techniques have allowed progress to be made in the understanding of how immune and stromal cells from the TME shape clinical outcome. With the growing use of therapies targeting subsets of the TME, they have proved particularly useful to gain insights into the functioning of these treatments. Together, the results presented in this review have provided advances toward a true precision medicine for cancer patients.

## Author contributions

FP wrote the manuscript, which was ammended and validated by all authors.

### Conflict of interest statement

The authors declare that the research was conducted in the absence of any commercial or financial relationships that could be construed as a potential conflict of interest. The reviewer FF and the handling Editor declared their shared affiliation.
